# Primary uveal lymphoma effectively managed with oral chlorambucil: a case report

**DOI:** 10.1186/1752-1947-7-173

**Published:** 2013-07-03

**Authors:** Iwona Rospond-Kubiak, Jarosław Kocięcki, Marcin Stopa

**Affiliations:** 1 Department of Ophthalmology, Poznań University of Medical Sciences, 1/2, Długa Street, Poznań, 61-848, Poland

**Keywords:** Chlorambucil, Primary uveal lymphoma

## Abstract

**Introduction:**

Ocular lymphomas account for five to 10 percent of all extra-nodal lymphomas. Primary uveal lymphoma is quite a rare entity and usually unilateral. We present a case of a primary uveal lymphoma with conjunctival and orbital extension, successfully managed with oral chlorambucil.

**Case presentation:**

A 71-year-old Caucasian man presented to our facility with visual loss in his only functioning eye (left). On clinical examination, we found a conjunctival lesion with a choroidal infiltration and a secondary retinal detachment. Ultrasound and magnetic resonance imaging studies revealed a choroidal tumour mass and two other lesions around the optic nerve. Results from an incisional biopsy revealed a low-grade B-cell lymphoma (CD20+, CD43+, bcl2+, CD3-). A diagnosis of primary uveal lymphoma was made. Our patient was started on a chemotherapy regime with no effect, and then oral chlorambucil was administered, with a relatively good result. At 10 months after the start of chlorambucil treatment, a best-corrected visual acuity of 0.4 was recorded, the choroidal mass had practically disappeared and the extra-ocular lesions had shrunk.

**Conclusions:**

In all, 61 to 80 cases of primary uveal lymphoma have already been described in the literature. Generally, it is an indolent tumor with a good prognosis. However, there are some reports of aggressive tumor behavior a few years after initial diagnosis (about eight percent of cases). Other treatment options are orbital irradiation at low doses (20 to 40 Gy) or steroid administration. This is the first documented report of the efficacy of oral chlorambucil in the treatment of primary uveal lymphoma.

## Introduction

Ophthalmic lymphomas account for five to 10 percent of all extra-nodal lymphomas [1,2]. The majority of orbital or conjunctival lymphomas are mucosa-associated lymphoid tissue (MALT) lymphomas. Primary uveal lymphoma “(‘infiltration lymphoïde uvéal’ [ILU])” is quite a rare entity, and is generally unilateral. In contrast, primary vitreoretinal lymphoma may be bilateral and very aggressive, with the prompt invasion of the central nervous system (CNS).

Here, we present a case of a primary uveal lymphoma with typical conjunctival and orbital extension successfully managed with oral chlorambucil.

## Case presentation

A 71-year-old Caucasian man was referred to our facility due to sudden visual deterioration in his only functional eye (left). His vision was 0.2 in the affected eye and hand movement only with the right eye, which had been amblyopic since his childhood.

**Figure 1:**
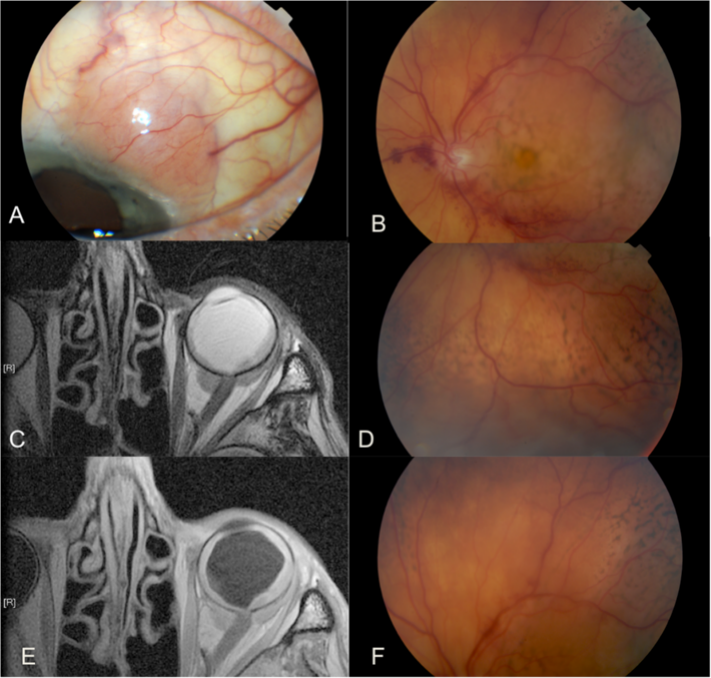
**Left eye at presentation. (A)** Conjunctival lesion in the superotemporal quadrant, **(B)** fundus of the left eye showing the posterior pole **(B)** and choroidal infiltration with secondary retinal detachment in the inferior (**D**) and superior (**F**) periphery. **(C,E)** Magnetic resonance imaging of the orbits revealing left choroidal tumor mass and two lesions around the optic nerve in T1-weighted and T2-weighted images, respectively.

Anterior segment biomicroscopy results revealed a left conjunctival lesion in the superotemporal quadrant. On fundoscopy we noted a choroidal infiltration with exudative retinal detachment (Figure 1A,B,D,F).

Ocular and orbital imaging results revealed a choroidal mass (thickening of chorioretinal layer extending for 2.5mm) and two retrobulbar lesions around the optic nerve (6.6×4.4mm and 5.8×3.3mm) (Figure 1C,D).

Incisional conjunctival biopsy results revealed a B-cell lymphoma (CD20+, CD43+, bcl2+, CD3-). Additional examinations (blood films, computed tomography (CT) scans of the abdomen and thorax, and bone marrow biopsy) excluded other systemic locations of disease.

Due to the clinical presentation in the absence of any systemic extension of the lymphoma, we made a final diagnosis of a primary uveal lymphoma, which is generally an indolent tumor with a good life prognosis.

**Figure 2:**
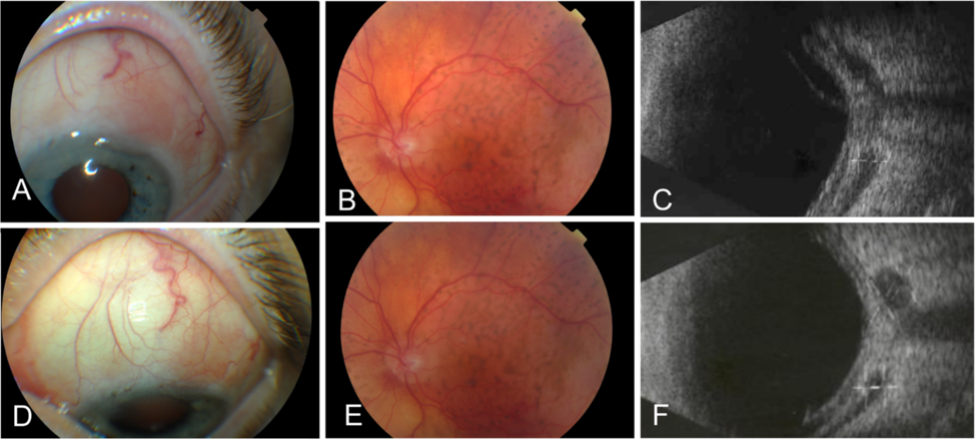
**The response to oral chlorambucil at four (A,B,C) and 10 (D,E,F) months after the onset of therapy.** The conjunctival lesion is diminished **(A,D),** with flattening of the choroid together with disappearance of secondary retinal detachment **(B,E) **and regression of the choroid mass and retrobulbar lesions on ultrasound **(C,F)**.

Our patient was referred for hemato-oncologic counseling and was initially treated with a systemic chemotherapy regime (CHOP, for ‘cyclophosphamide, hydroxydaunorubicin, Oncovin® (vincristine), prednisone’) with no effect; oral chlorambucil was then administered, with a good response (Figure 2).

At 10 months after the onset of chlorambucil therapy, the best-corrected visual acuity in his left eye had improved to 0.4, the choroidal mass had practically disappeared and the retrobulbar lesions had regressed. At 12-month follow-up no systemic extension was noted.

## Discussion

Primary uveal lymphoma is a rare pathology; there have only been about 61 to 80 cases reported in the literature [1,2]. The first report was from Trebenstein in 1920 [1,2].

This condition was initially considered as an inflammatory process rather than a proliferation of lymphocytes and histiocytes of low malignancy that starts in the choroidal tissue, as it is recognized as nowadays. Conjunctival or orbital involvement in these cases is secondary [2]. However, recently a report of secondary uveal infiltration by a MALT lymphoma originating from the conjunctiva has been published [3].

Differentiating between these two types of lymphoma can be sometimes difficult since the immunohistochemical or histopathological characteristics of intra-ocular and extra-ocular lesions can differ in the same patient. This limits the reliability of excisional biopsy results [2]. Diagnosis of primary uveal lymphoma should be made after a histopathological examination of a lesion sample in the absence of other systemic locations of lymphoma at presentation, as was done in our patient’s case [1].

ILU is generally believed to be an indolent tumor with a good life expectancy. However, it can become aggressive and some systemic extension can appear a couple of years after initial diagnosis (this occurs in about eight percent of cases) [3]. Other treatment options proposed in the literature include orbital irradiation (20 to 40 Gy) or steroids [1]. In our patient’s case, his only functional eye was affected and there was a risk of subsequent radiation side effects, since the tumor was covering most of the choroid.

Chlorambucil (Leukeran®) is a nitrogen mustard alkylating agent mainly used in chronic lymphocytic leukemia [4]. Its use in other systemic indolent lymphoma is widely known, especially in older patients; with regard to primary uveal lymphoma this is the first documented report of its efficacy.

## Conclusions

In summary, to the best of our knowledge this is the first documented report of efficacy of oral chlorambucil in treatment of a case of ILU.

## Consent

Written informed consent was obtained from the patient for publication of this report and any accompanying images. A copy of the written consent is available for review by the Editor-in-Chief of this journal.

## Competing interests

The authors declare that they have no competing interests.

## Authors’ contributions

IRK was responsible for the clinical management of our patient and preparation of the first draft of the manuscript. JK supervised the treatment of our patient and revised the manuscript. MS performed the literature search and revised the manuscript. The manuscript was prepared without the help of professional medical writers. All authors approved the final version of the manuscript.
